# National Health Week

**Published:** 1920-04-17

**Authors:** 


### THE PUBLIC WELL-BEING.

### NATIONAL HEALTH WEEK.

### Self-Help the Dominant Idea.

The first week in May has been reserved for the celebration of National Health Week, and already there are indications that the movement will rapidly cover the ground lost through the necessary interruption due to the war. We are informed by the Health Week Committee, appointed by the Royal Sanitary Institute, that no fewer than 300 local authorities throughout the country are in correspondence with those responsible for organising Health Week, and the different localities are encouraged to make use of the occasion to develop on their own lines measures for informing and awakening the public mind. The object is to focus public attention for one week in the year on matters of health, and to arouse that sense of personal responsibility for health, without which, it is wisely observed, all public work by the Government and local authorities must fall far short of its aims.

It is the considered opinion of medical officers of health and other experts, that the improvement of health is fast approaching its limit on existing lines. No further general advance, it is declared, can be expected until the people themselves co-operate more closely in the work of the public service. In short, the health of a community depends as much on the man himself as on his surroundings, and on personal hygiene and cleanliness as upon the work of the sanitary authorities. The dominant idea, therefore, for this year is the importance of self-help, and the consideration of what each individual can do for himself and his neighbour in securing a healthy life. Programmes under consideration in various centres include lectures on subjects ranging from street scavenging to microbes, cinema shows, baby competitions, cookery demonstrations, health exhibitions, sermons, and competitive essays for scholars in day and Sunday schools. Considerable impetus has been given to the movement by the keen personal interest displayed by the King and Queen, both of whom are patrons.

The movement known as Health Week was instituted in 1912, when it was observed by some eighty to one hundred authorities in the United Kingdom. This year the number has largely increased, and the movement also has the advantage of the backing of the Ministry of Health. Care will have to be taken that some brightness and interest is given to the subjects which are handled by the local committees, and no doubt the promoters of the scheme will bear this in mind in their dealings with them. They utter a warning word against anything in the nature of sensationalism, and, of course, it is not necessary to be sensational in order to attract interest. There are many instances of the attractive way in which experts in health matters have been able to present to the public the most important lessons. This Health Week is a great opportunity for the health propagandist to exercise not only his enthusiasm, but his ingenuity, and it is hoped that no effort will be spared to make all the arrangements widely known. Have the organising committee considered a proposal made in these columns on several occasions, that there should be a "Safety-First" campaign in relation to health? The Health Week seems to be an admirable opportunity for an intensive campaign of that description. Backed up by local authorities, and supported by all responsible people, it ought not to be an expensive undertaking, and it seems an excellent chance for such an experiment.

Viscountess Rhondda, who may be said to have an hereditary interest in public health, said a short while ago that the Ministry of Health, although it had at its disposal some of the best brains the Civil Service could produce, would not be able to act as it should without a strong public opinion behind it. Health was not a cheap commodity, although it was cheap at the price. She asked for a strong and enlightened public opinion to support the Ministry in its endeavours. If they could only make it possible to get the best possible standard of health in the country, they would be conferring a most precious gift upon the generations to come. It is precisely in this spirit that the Health Week ought to be, and is being, promoted.

